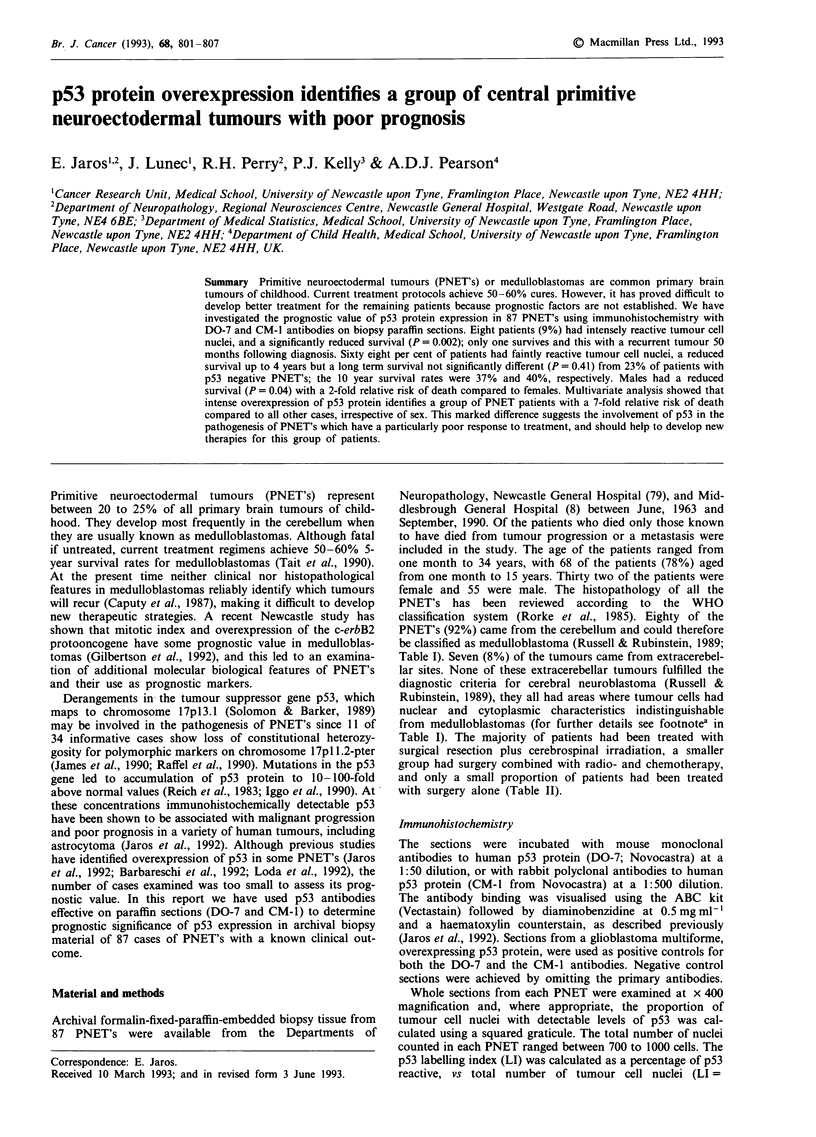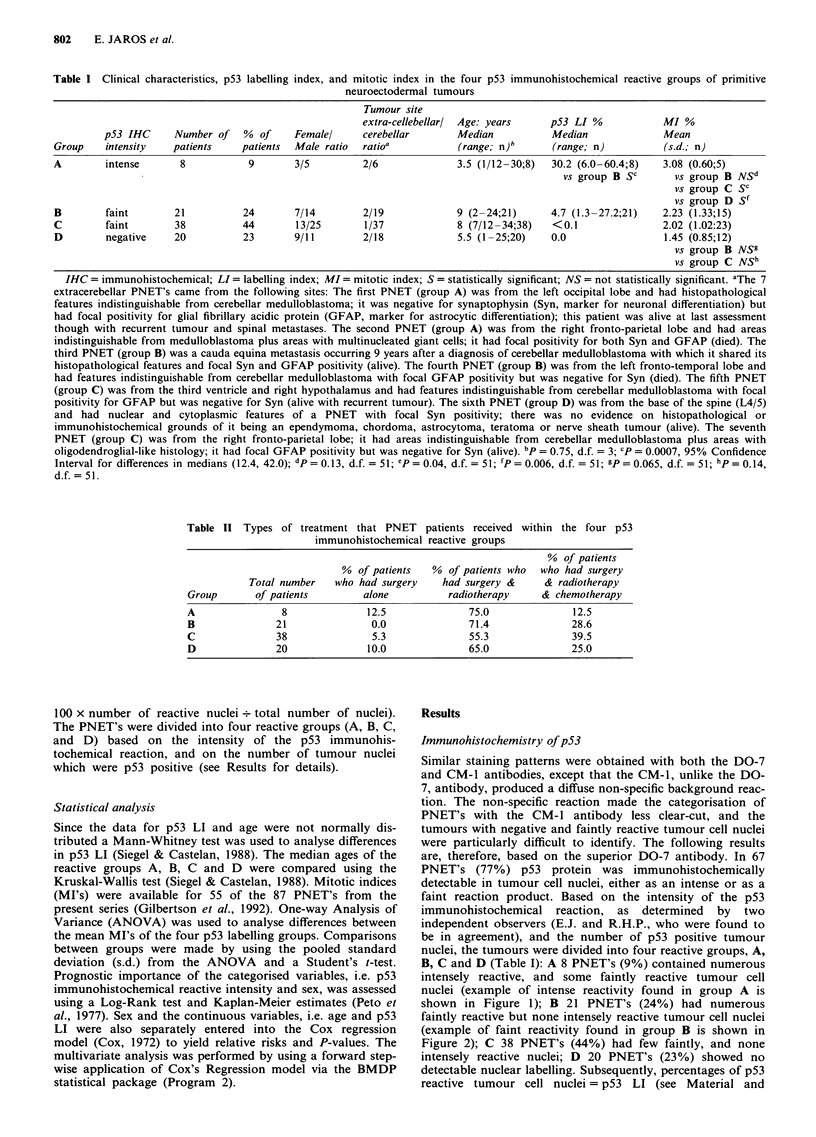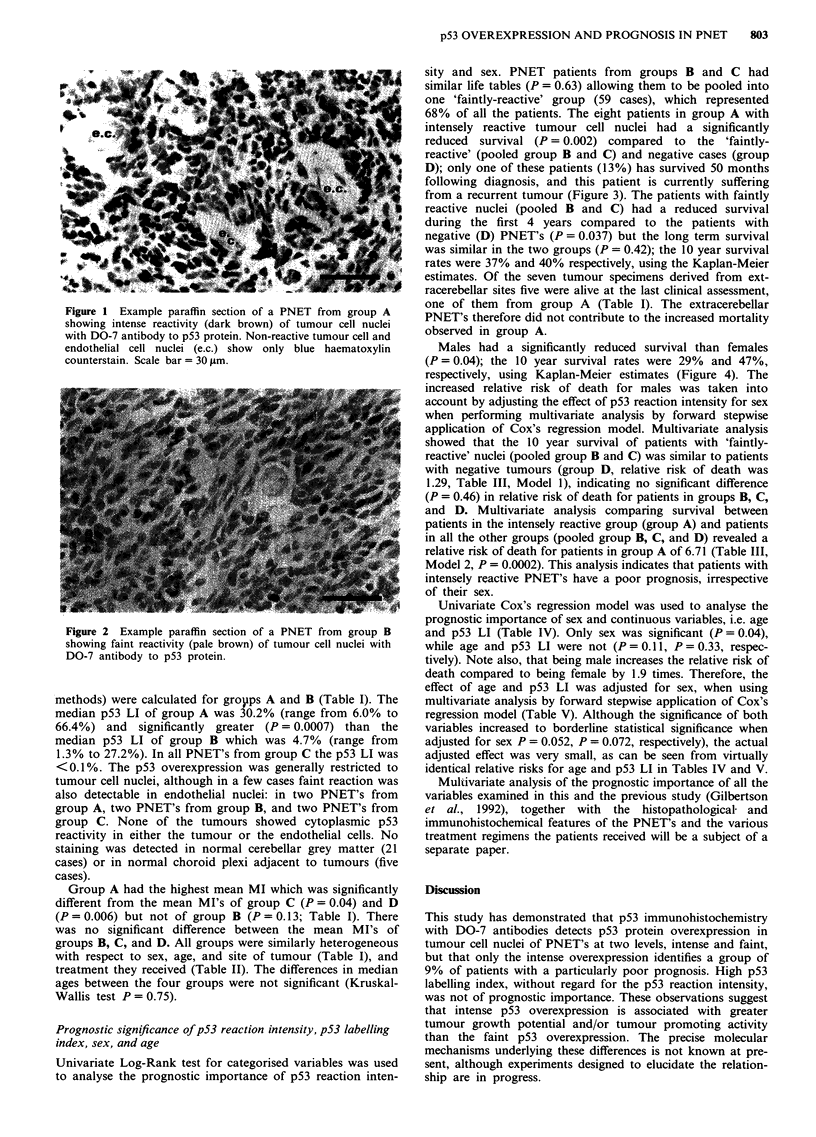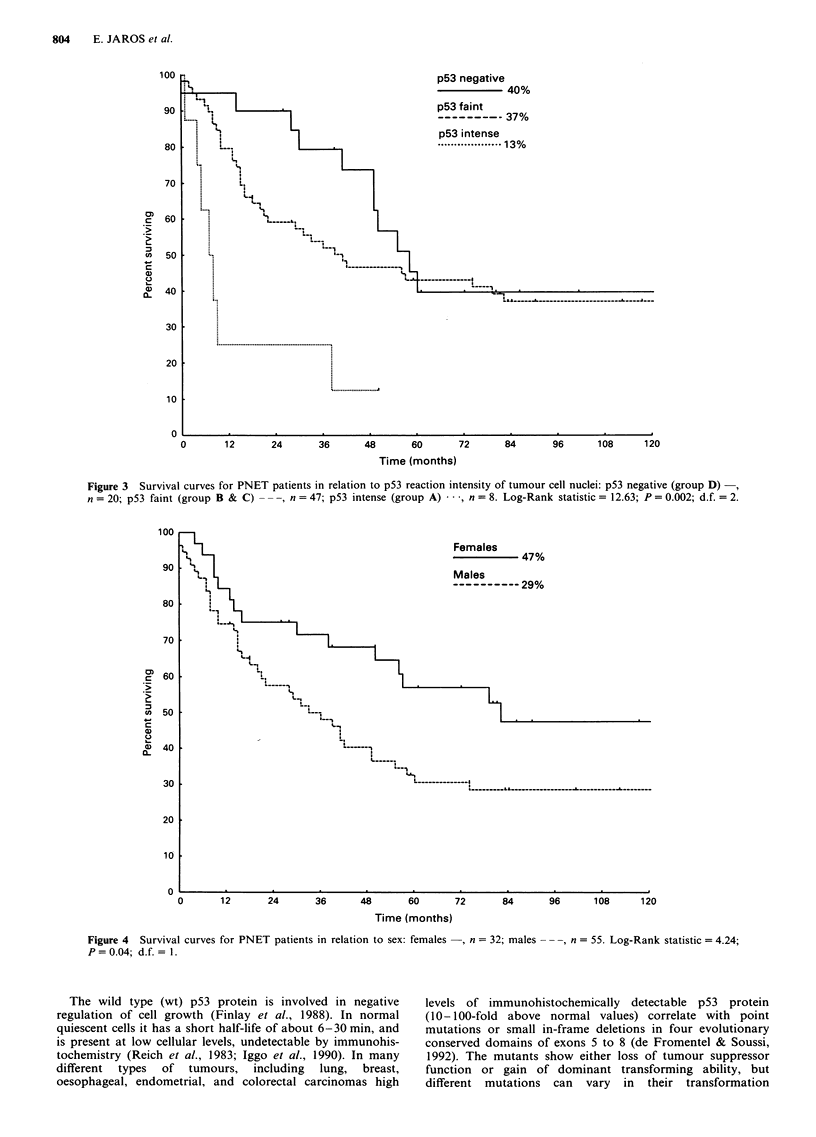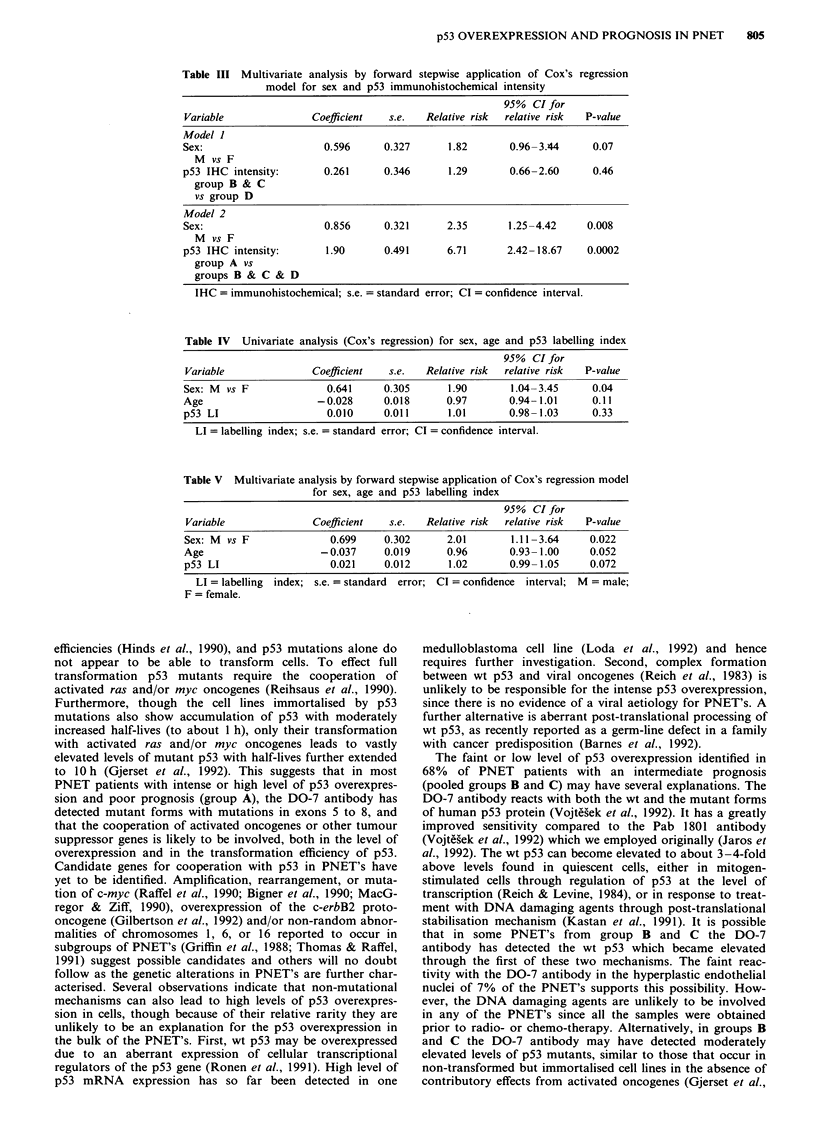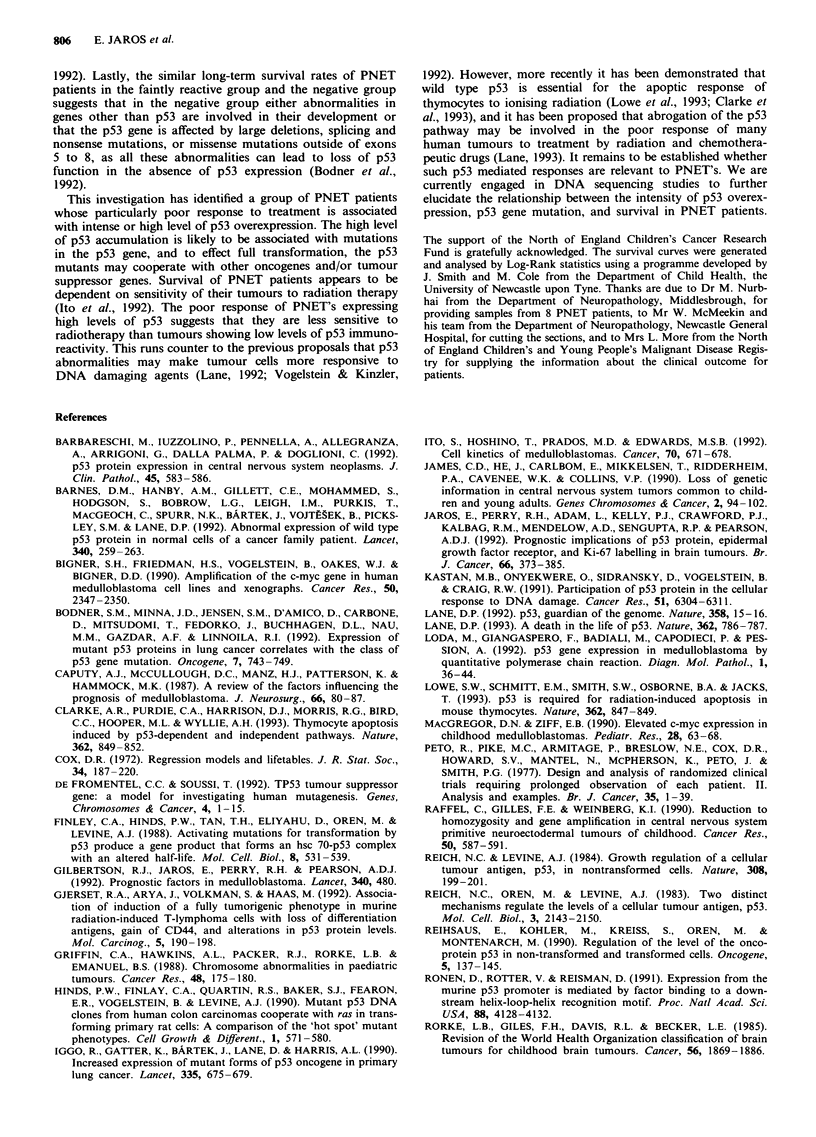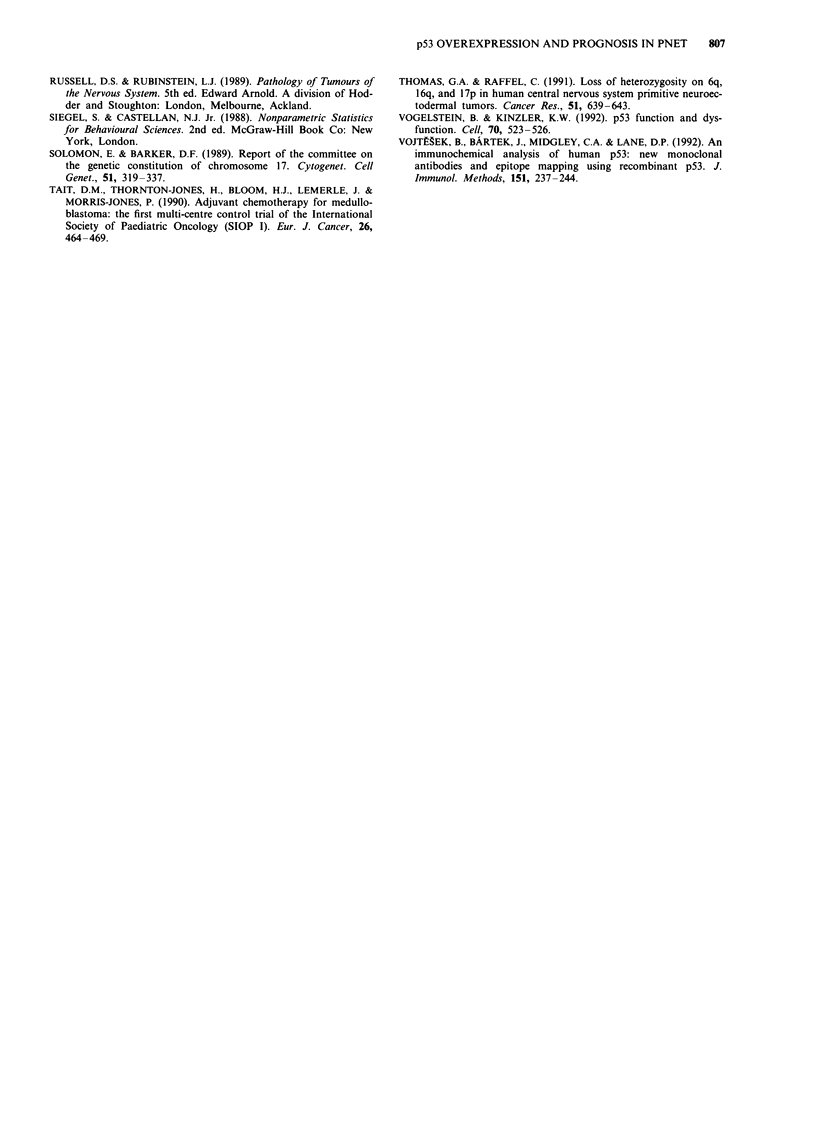# p53 protein overexpression identifies a group of central primitive neuroectodermal tumours with poor prognosis.

**DOI:** 10.1038/bjc.1993.431

**Published:** 1993-10

**Authors:** E. Jaros, J. Lunec, R. H. Perry, P. J. Kelly, A. D. Pearson

**Affiliations:** Cancer Research Unit, Medical School, University of Newcastle upon Tyne, UK.

## Abstract

**Images:**


Br. J. Cancer (1993), 68, 801-807 © Macmillan Press Ltd., 1993

### p53 protein overexpression identifies a group of central primitive neuroectodermal tumours with poor prognosis

E. Jaros[1,2], J. Lunec[1], R.H. Perry[2], P.J. Kelly[3] & A.D.J. Pearson[4]

[1]*Cancer Research Unit, Medical School, University of Newcastle upon Tyne, Framlington Place, Newcastle upon Tyne, NE2 4HH;* [2]*Department of Neuropathology, Regional Neurosciences Centre, Newcastle General Hospital, Westgate Road, Newcastle upon Tyne, NE4 6BE;* [3]*Department of Medical Statistics, Medical School, University of Newcastle upon Tyne, Framlington Place, Newcastle upon Tyne, NE2 4HH;* [4]*Department of Child Health, Medical School, University of Newcastle upon Tyne, Framlington Place, Newcastle upon Tyne, NE2 4HH, UK.*

**Summary** Primitive neuroectodermal tumours (PNET's) or medulloblastomas are common primary brain tumours of childhood. Current treatment protocols achieve 50–60% cures. However, it has proved difficult to develop better treatment for the remaining patients because prognostic factors are not established. We have investigated the prognostic value of p53 protein expression in 87 PNET's using immunohistochemistry with DO-7 and CM-1 antibodies on biopsy paraffin sections. Eight patients (9%) had intensely reactive tumour cell nuclei, and a significantly reduced survival ($P = 0.002$); only one survives and this with a recurrent tumour 50 months following diagnosis. Sixty eight per cent of patients had faintly reactive tumour cell nuclei, a reduced survival up to 4 years but a long term survival not significantly different ($P = 0.41$) from 23% of patients with p53 negative PNET's; the 10 year survival rates were 37% and 40%, respectively. Males had a reduced survival ($P = 0.04$) with a 2-fold relative risk of death compared to females. Multivariate analysis showed that intense overexpression of p53 protein identifies a group of PNET patients with a 7-fold relative risk of death compared to all other cases, irrespective of sex. This marked difference suggests the involvement of p53 in the pathogenesis of PNET's which have a particularly poor response to treatment, and should help to develop new therapies for this group of patients.

Primitive neuroectodermal tumours (PNET's) represent between 20 to 25% of all primary brain tumours of childhood. They develop most frequently in the cerebellum when they are usually known as medulloblastomas. Although fatal if untreated, current treatment regimens achieve 50–60% 5-year survival rates for medulloblastomas (Tait *et al.*, 1990). At the present time neither clinical nor histopathological features in medulloblastomas reliably identify which tumours will recur (Caputy *et al.*, 1987), making it difficult to develop new therapeutic strategies. A recent Newcastle study has shown that mitotic index and overexpression of the c-*erb*B2 protooncogene have some prognostic value in medulloblastomas (Gilbertson *et al.*, 1992), and this led to an examination of additional molecular biological features of PNET's and their use as prognostic markers.

Derangements in the tumour suppressor gene p53, which maps to chromosome 17p13.1 (Solomon & Barker, 1989) may be involved in the pathogenesis of PNET's since 11 of 34 informative cases show loss of constitutional heterozygosity for polymorphic markers on chromosome 17p11.2-pter (James *et al.*, 1990; Raffel *et al.*, 1990). Mutations in the p53 gene led to accumulation of p53 protein to 10–100-fold above normal values (Reich *et al.*, 1983; Iggo *et al.*, 1990). At these concentrations immunohistochemically detectable p53 have been shown to be associated with malignant progression and poor prognosis in a variety of human tumours, including astrocytoma (Jaros *et al.*, 1992). Although previous studies have identified overexpression of p53 in some PNET's (Jaros *et al.*, 1992; Barbareschi *et al.*, 1992; Loda *et al.*, 1992), the number of cases examined was too small to assess its prognostic value. In this report we have used p53 antibodies effective on paraffin sections (DO-7 and CM-1) to determine prognostic significance of p53 expression in archival biopsy material of 87 cases of PNET's with a known clinical outcome.

### Material and methods

Archival formalin-fixed-paraffin-embedded biopsy tissue from 87 PNET's were available from the Departments of Neuropathology, Newcastle General Hospital (79), and Middlesbrough General Hospital (8) between June, 1963 and September, 1990. Of the patients who died only those known to have died from tumour progression or a metastasis were included in the study. The age of the patients ranged from one month to 34 years, with 68 of the patients (78%) aged from one month to 15 years. Thirty two of the patients were female and 55 were male. The histopathology of all the PNET's has been reviewed according to the WHO classification system (Rorke *et al.*, 1985). Eighty of the PNET's (92%) came from the cerebellum and could therefore be classified as medulloblastoma (Russell & Rubinstein, 1989; Table I). Seven (8%) of the tumours came from extracerebellar sites. None of these extracerebellar tumours fulfilled the diagnostic criteria for cerebral neuroblastoma (Russell & Rubinstein, 1989), they all had areas where tumour cells had nuclear and cytoplasmic characteristics indistinguishable from medulloblastomas (for further details see footnote[a] in Table I). The majority of patients had been treated with surgical resection plus cerebrospinal irradiation, a smaller group had surgery combined with radio- and chemotherapy, and only a small proportion of patients had been treated with surgery alone (Table II).

### Immunohistochemistry

The sections were incubated with mouse monoclonal antibodies to human p53 protein (DO-7; Novocastra) at a 1:50 dilution, or with rabbit polyclonal antibodies to human p53 protein (CM-1 from Novocastra) at a 1:500 dilution. The antibody binding was visualised using the ABC kit (Vectastain) followed by diaminobenzidine at $0.5 \, mg \, ml^{-1}$ and a haematoxylin counterstain, as described previously (Jaros *et al.*, 1992). Sections from a glioblastoma multiforme, overexpressing p53 protein, were used as positive controls for both the DO-7 and the CM-1 antibodies. Negative control sections were achieved by omitting the primary antibodies.

Whole sections from each PNET were examined at × 400 magnification and, where appropriate, the proportion of tumour cell nuclei with detectable levels of p53 was calculated using a squared graticule. The total number of nuclei counted in each PNET ranged between 700 to 1000 cells. The p53 labelling index (LI) was calculated as a percentage of p53 reactive, *vs* total number of tumour cell nuclei (LI =

Correspondence: E. Jaros.
Received 10 March 1993; and in revised form 3 June 1993.

**Table I** Clinical characteristics, p53 labelling index, and mitotic index in the four p53 immunohistochemical reactive groups of primitive neuroectodermal tumours

| Group | p53 IHC intensity | Number of patients | % of patients | Female/ Male ratio | Tumour site extra-cellebellar/ cerebellar ratio[a] | Age: years Median (range; n)[b] | p53 LI % Median (range; n) | MI % Mean (s.d.; n) |
|---|---|---|---|---|---|---|---|---|
| **A** | intense | 8 | 9 | 3/5 | 2/6 | 3.5 (1/12–30;8) | 30.2 (6.0–60.4;8) *vs* group **B** *S*[c] | 3.08 (0.60;5) *vs* group **B** *NS*[d] *vs* group **C** *S*[e] *vs* group **D** *S*[f] |
| **B** | faint | 21 | 24 | 7/14 | 2/19 | 9 (2–24;21) | 4.7 (1.3–27.2;21) | 2.23 (1.33;15) |
| **C** | faint | 38 | 44 | 13/25 | 1/37 | 8 (7/12–34;38) | <0.1 | 2.02 (1.02:23) |
| **D** | negative | 20 | 23 | 9/11 | 2/18 | 5.5 (1–25;20) | 0.0 | 1.45 (0.85;12) *vs* group **B** *NS*[g] *vs* group **C** *NS*[h] |

*IHC* = immunohistochemical; *LI* = labelling index; *MI* = mitotic index; *S* = statistically significant; *NS* = not statistically significant. [a]The 7 extracerebellar PNET's came from the following sites: The first PNET (group **A**) was from the left occipital lobe and had histopathological features indistinguishable from cerebellar medulloblastoma; it was negative for synaptophysin (Syn, marker for neuronal differentiation) but had focal positivity for glial fibrillary acidic protein (GFAP, marker for astrocytic differentiation); this patient was alive at last assessment though with recurrent tumour and spinal metastases. The second PNET (group **A**) was from the right fronto-parietal lobe and had areas indistinguishable from medulloblastoma plus areas with multinucleated giant cells; it had focal positivity for both Syn and GFAP (died). The third PNET (group **B**) was a cauda equina metastasis occurring 9 years after a diagnosis of cerebellar medulloblastoma with which it shared its histopathological features and focal Syn and GFAP positivity (alive). The fourth PNET (group **B**) was from the left fronto-temporal lobe and had features indistinguishable from cerebellar medulloblastoma with focal GFAP positivity but was negative for Syn (died). The fifth PNET (group **C**) was from the third ventricle and right hypothalamus and had features indistinguishable from cerebellar medulloblastoma with focal positivity for GFAP but was negative for Syn (alive with recurrent tumour). The sixth PNET (group **D**) was from the base of the spine (L4/5) and had nuclear and cytoplasmic features of a PNET with focal Syn positivity; there was no evidence on histopathological or immunohistochemical grounds of it being an ependymoma, chordoma, astrocytoma, teratoma or nerve sheath tumour (alive). The seventh PNET (group **C**) was from the right fronto-parietal lobe; it had areas indistinguishable from cerebellar medulloblastoma plus areas with oligodendroglial-like histology; it had focal GFAP positivity but was negative for Syn (alive). [b]$P = 0.75$, d.f. = 3; [c]$P = 0.0007$, 95% Confidence Interval for differences in medians (12.4, 42.0); [d]$P = 0.13$, d.f. = 51; [e]$P = 0.04$, d.f. = 51; [f]$P = 0.006$, d.f. = 51; [g]$P = 0.065$, d.f. = 51; [h]$P = 0.14$, d.f. = 51.

**Table II** Types of treatment that PNET patients received within the four p53 immunohistochemical reactive groups

| Group | Total number of patients | % of patients who had surgery alone | % of patients who had surgery & radiotherapy | % of patients who had surgery & radiotherapy & chemotherapy |
|---|---|---|---|---|
| **A** | 8 | 12.5 | 75.0 | 12.5 |
| **B** | 21 | 0.0 | 71.4 | 28.6 |
| **C** | 38 | 5.3 | 55.3 | 39.5 |
| **D** | 20 | 10.0 | 65.0 | 25.0 |

100 × number of reactive nuclei ÷ total number of nuclei). The PNET's were divided into four reactive groups (A, B, C, and D) based on the intensity of the p53 immunohistochemical reaction, and on the number of tumour nuclei which were p53 positive (see Results for details).

*Statistical analysis*

Since the data for p53 LI and age were not normally distributed a Mann-Whitney test was used to analyse differences in p53 LI (Siegel & Castelan, 1988). The median ages of the reactive groups A, B, C and D were compared using the Kruskal-Wallis test (Siegel & Castelan, 1988). Mitotic indices (MI's) were available for 55 of the 87 PNET's from the present series (Gilbertson *et al.*, 1992). One-way Analysis of Variance (ANOVA) was used to analyse differences between the mean MI's of the four p53 labelling groups. Comparisons between groups were made by using the pooled standard deviation (s.d.) from the ANOVA and a Student's *t*-test. Prognostic importance of the categorised variables, i.e. p53 immunohistochemical reactive intensity and sex, was assessed using a Log-Rank test and Kaplan-Meier estimates (Peto *et al.*, 1977). Sex and the continuous variables, i.e. age and p53 LI were also separately entered into the Cox regression model (Cox, 1972) to yield relative risks and *P*-values. The multivariate analysis was performed by using a forward stepwise application of Cox's Regression model via the BMDP statistical package (Program 2).

### Results

*Immunohistochemistry of p53*

Similar staining patterns were obtained with both the DO-7 and CM-1 antibodies, except that the CM-1, unlike the DO-7, antibody, produced a diffuse non-specific background reaction. The non-specific reaction made the categorisation of PNET's with the CM-1 antibody less clear-cut, and the tumours with negative and faintly reactive tumour cell nuclei were particularly difficult to identify. The following results are, therefore, based on the superior DO-7 antibody. In 67 PNET's (77%) p53 protein was immunohistochemically detectable in tumour cell nuclei, either as an intense or as a faint reaction product. Based on the intensity of the p53 immunohistochemical reaction, as determined by two independent observers (E.J. and R.H.P., who were found to be in agreement), and the number of p53 positive tumour nuclei, the tumours were divided into four reactive groups, **A**, **B**, **C** and **D** (Table I): **A** 8 PNET's (9%) contained numerous intensely reactive, and some faintly reactive tumour cell nuclei (example of intense reactivity found in group **A** is shown in Figure 1); **B** 21 PNET's (24%) had numerous faintly reactive but none intensely reactive tumour cell nuclei (example of faint reactivity found in group **B** is shown in Figure 2); **C** 38 PNET's (44%) had few faintly, and none intensely reactive nuclei; **D** 20 PNET's (23%) showed no detectable nuclear labelling. Subsequently, percentages of p53 reactive tumour cell nuclei = p53 LI (see Material and

**Figure 1** Example paraffin section of a PNET from group **A** showing intense reactivity (dark brown) of tumour cell nuclei with DO-7 antibody to p53 protein. Non-reactive tumour cell and endothelial cell nuclei (e.c.) show only blue haematoxylin counterstain. Scale bar = 30 μm.

**Figure 2** Example paraffin section of a PNET from group **B** showing faint reactivity (pale brown) of tumour cell nuclei with DO-7 antibody to p53 protein.

methods) were calculated for groups **A** and **B** (Table I). The median p53 LI of group **A** was 30.2% (range from 6.0% to 66.4%) and significantly greater ($P = 0.0007$) than the median p53 LI of group **B** which was 4.7% (range from 1.3% to 27.2%). In all PNET's from group **C** the p53 LI was <0.1%. The p53 overexpression was generally restricted to tumour cell nuclei, although in a few cases faint reaction was also detectable in endothelial nuclei: in two PNET's from group **A**, two PNET's from group **B**, and two PNET's from group **C**. None of the tumours showed cytoplasmic p53 reactivity in either the tumour or the endothelial cells. No staining was detected in normal cerebellar grey matter (21 cases) or in normal choroid plexi adjacent to tumours (five cases).

Group **A** had the highest mean MI which was significantly different from the mean MI's of group **C** ($P = 0.04$) and **D** ($P = 0.006$) but not of group **B** ($P = 0.13$; Table I). There was no significant difference between the mean MI's of groups **B, C,** and **D.** All groups were similarly heterogeneous with respect to sex, age, and site of tumour (Table I), and treatment they received (Table II). The differences in median ages between the four groups were not significant (Kruskal-Wallis test $P = 0.75$).

*Prognostic significance of p53 reaction intensity, p53 labelling index, sex, and age*

Univariate Log-Rank test for categorised variables was used to analyse the prognostic importance of p53 reaction intensity and sex. PNET patients from groups **B** and **C** had similar life tables ($P = 0.63$) allowing them to be pooled into one 'faintly-reactive' group (59 cases), which represented 68% of all the patients. The eight patients in group **A** with intensely reactive tumour cell nuclei had a significantly reduced survival ($P = 0.002$) compared to the 'faintly-reactive' (pooled group **B** and **C**) and negative cases (group **D**); only one of these patients (13%) has survived 50 months following diagnosis, and this patient is currently suffering from a recurrent tumour (Figure 3). The patients with faintly reactive nuclei (pooled **B** and **C**) had a reduced survival during the first 4 years compared to the patients with negative (**D**) PNET's ($P = 0.037$) but the long term survival was similar in the two groups ($P = 0.42$); the 10 year survival rates were 37% and 40% respectively, using the Kaplan-Meier estimates. Of the seven tumour specimens derived from extracerebellar sites five were alive at the last clinical assessment, one of them from group A (Table I). The extracerebellar PNET's therefore did not contribute to the increased mortality observed in group **A**.

Males had a significantly reduced survival than females ($P = 0.04$); the 10 year survival rates were 29% and 47%, respectively, using Kaplan-Meier estimates (Figure 4). The increased relative risk of death for males was taken into account by adjusting the effect of p53 reaction intensity for sex when performing multivariate analysis by forward stepwise application of Cox's regression model. Multivariate analysis showed that the 10 year survival of patients with 'faintly-reactive' nuclei (pooled group **B** and **C**) was similar to patients with negative tumours (group **D**, relative risk of death was 1.29, Table III, Model 1), indicating no significant difference ($P = 0.46$) in relative risk of death for patients in groups **B, C,** and **D.** Multivariate analysis comparing survival between patients in the intensely reactive group (group **A**) and patients in all the other groups (pooled group **B, C,** and **D**) revealed a relative risk of death for patients in group **A** of 6.71 (Table III, Model 2, $P = 0.0002$). This analysis indicates that patients with intensely reactive PNET's have a poor prognosis, irrespective of their sex.

Univariate Cox's regression model was used to analyse the prognostic importance of sex and continuous variables, i.e. age and p53 LI (Table IV). Only sex was significant ($P = 0.04$), while age and p53 LI were not ($P = 0.11$, $P = 0.33$, respectively). Note also, that being male increases the relative risk of death compared to being female by 1.9 times. Therefore, the effect of age and p53 LI was adjusted for sex, when using multivariate analysis by forward stepwise application of Cox's regression model (Table V). Although the significance of both variables increased to borderline statistical significance when adjusted for sex $P = 0.052$, $P = 0.072$, respectively), the actual adjusted effect was very small, as can be seen from virtually identical relative risks for age and p53 LI in Tables IV and V.

Multivariate analysis of the prognostic importance of all the variables examined in this and the previous study (Gilbertson *et al.*, 1992), together with the histopathological and immunohistochemical features of the PNET's and the various treatment regimens the patients received will be a subject of a separate paper.

### Discussion

This study has demonstrated that p53 immunohistochemistry with DO-7 antibodies detects p53 protein overexpression in tumour cell nuclei of PNET's at two levels, intense and faint, but that only the intense overexpression identifies a group of 9% of patients with a particularly poor prognosis. High p53 labelling index, without regard for the p53 reaction intensity, was not of prognostic importance. These observations suggest that intense p53 overexpression is associated with greater tumour growth potential and/or tumour promoting activity than the faint p53 overexpression. The precise molecular mechanisms underlying these differences is not known at present, although experiments designed to elucidate the relationship are in progress.

**Figure 3** Survival curves for PNET patients in relation to p53 reaction intensity of tumour cell nuclei: p53 negative (group **D**) —, $n = 20$; p53 faint (group **B** & **C**) - - -, $n = 47$; p53 intense (group **A**) · · ·, $n = 8$. Log-Rank statistic = 12.63; $P = 0.002$; d.f. = 2.

**Figure 4** Survival curves for PNET patients in relation to sex: females —, $n = 32$; males - - -, $n = 55$. Log-Rank statistic = 4.24; $P = 0.04$; d.f. = 1.

The wild type (wt) p53 protein is involved in negative regulation of cell growth (Finlay *et al.*, 1988). In normal quiescent cells it has a short half-life of about 6–30 min, and is present at low cellular levels, undetectable by immunohistochemistry (Reich *et al.*, 1983; Iggo *et al.*, 1990). In many different types of tumours, including lung, breast, oesophageal, endometrial, and colorectal carcinomas high levels of immunohistochemically detectable p53 protein (10–100-fold above normal values) correlate with point mutations or small in-frame deletions in four evolutionary conserved domains of exons 5 to 8 (de Fromentel & Soussi, 1992). The mutants show either loss of tumour suppressor function or gain of dominant transforming ability, but different mutations can vary in their transformation

**Table III** Multivariate analysis by forward stepwise application of Cox's regression model for sex and p53 immunohistochemical intensity

| *Variable* | *Coefficient* | *s.e.* | *Relative risk* | *95% CI for relative risk* | P-*value* |
|---|---|---|---|---|---|
| *Model 1* | | | | | |
| Sex: M *vs* F | 0.596 | 0.327 | 1.82 | 0.96–3.44 | 0.07 |
| p53 IHC intensity: group **B** & **C** *vs* group **D** | 0.261 | 0.346 | 1.29 | 0.66–2.60 | 0.46 |
| *Model 2* | | | | | |
| Sex: M *vs* F | 0.856 | 0.321 | 2.35 | 1.25–4.42 | 0.008 |
| p53 IHC intensity: group **A** *vs* groups **B** & **C** & **D** | 1.90 | 0.491 | 6.71 | 2.42–18.67 | 0.0002 |

IHC = immunohistochemical; s.e. = standard error; CI = confidence interval.

**Table IV** Univariate analysis (Cox's regression) for sex, age and p53 labelling index

| *Variable* | *Coefficient* | *s.e.* | *Relative risk* | *95% CI for relative risk* | P-*value* |
|---|---|---|---|---|---|
| Sex: M *vs* F | 0.641 | 0.305 | 1.90 | 1.04–3.45 | 0.04 |
| Age | –0.028 | 0.018 | 0.97 | 0.94–1.01 | 0.11 |
| p53 LI | 0.010 | 0.011 | 1.01 | 0.98–1.03 | 0.33 |

LI = labelling index; s.e. = standard error; CI = confidence interval.

**Table V** Multivariate analysis by forward stepwise application of Cox's regression model for sex, age and p53 labelling index

| *Variable* | *Coefficient* | *s.e.* | *Relative risk* | *95% CI for relative risk* | P-*value* |
|---|---|---|---|---|---|
| Sex: M *vs* F | 0.699 | 0.302 | 2.01 | 1.11–3.64 | 0.022 |
| Age | –0.037 | 0.019 | 0.96 | 0.93–1.00 | 0.052 |
| p53 LI | 0.021 | 0.012 | 1.02 | 0.99–1.05 | 0.072 |

LI = labelling index; s.e. = standard error; CI = confidence interval; M = male; F = female.

efficiencies (Hinds *et al.*, 1990), and p53 mutations alone do not appear to be able to transform cells. To effect full transformation p53 mutants require the cooperation of activated *ras* and/or *myc* oncogenes (Reihsaus *et al.*, 1990). Furthermore, though the cell lines immortalised by p53 mutations also show accumulation of p53 with moderately increased half-lives (to about 1 h), only their transformation with activated *ras* and/or *myc* oncogenes leads to vastly elevated levels of mutant p53 with half-lives further extended to 10 h (Gjerset *et al.*, 1992). This suggests that in most PNET patients with intense or high level of p53 overexpression and poor prognosis (group **A**), the DO-7 antibody has detected mutant forms with mutations in exons 5 to 8, and that the cooperation of activated oncogenes or other tumour suppressor genes is likely to be involved, both in the level of overexpression and in the transformation efficiency of p53. Candidate genes for cooperation with p53 in PNET's have yet to be identified. Amplification, rearrangement, or mutation of c-*myc* (Raffel *et al.*, 1990; Bigner *et al.*, 1990; MacGregor & Ziff, 1990), overexpression of the c-*erb*B2 proto-oncogene (Gilbertson *et al.*, 1992) and/or non-random abnormalities of chromosomes 1, 6, or 16 reported to occur in subgroups of PNET's (Griffin *et al.*, 1988; Thomas & Raffel, 1991) suggest possible candidates and others will no doubt follow as the genetic alterations in PNET's are further characterised. Several observations indicate that non-mutational mechanisms can also lead to high levels of p53 overexpression in cells, though because of their relative rarity they are unlikely to be an explanation for the p53 overexpression in the bulk of the PNET's. First, wt p53 may be overexpressed due to an aberrant expression of cellular transcriptional regulators of the p53 gene (Ronen *et al.*, 1991). High level of p53 mRNA expression has so far been detected in one medulloblastoma cell line (Loda *et al.*, 1992) and hence requires further investigation. Second, complex formation between wt p53 and viral oncogenes (Reich *et al.*, 1983) is unlikely to be responsible for the intense p53 overexpression, since there is no evidence of a viral aetiology for PNET's. A further alternative is aberrant post-translational processing of wt p53, as recently reported as a germ-line defect in a family with cancer predisposition (Barnes *et al.*, 1992).

The faint or low level of p53 overexpression identified in 68% of PNET patients with an intermediate prognosis (pooled groups **B** and **C**) may have several explanations. The DO-7 antibody reacts with both the wt and the mutant forms of human p53 protein (Vojtěšek *et al.*, 1992). It has a greatly improved sensitivity compared to the Pab 1801 antibody (Vojtěšek *et al.*, 1992) which we employed originally (Jaros *et al.*, 1992). The wt p53 can become elevated to about 3–4-fold above levels found in quiescent cells, either in mitogen-stimulated cells through regulation of p53 at the level of transcription (Reich & Levine, 1984), or in response to treatment with DNA damaging agents through post-translational stabilisation mechanism (Kastan *et al.*, 1991). It is possible that in some PNET's from group **B** and **C** the DO-7 antibody has detected the wt p53 which became elevated through the first of these two mechanisms. The faint reactivity with the DO-7 antibody in the hyperplastic endothelial nuclei of 7% of the PNET's supports this possibility. However, the DNA damaging agents are unlikely to be involved in any of the PNET's since all the samples were obtained prior to radio- or chemo-therapy. Alternatively, in groups **B** and **C** the DO-7 antibody may have detected moderately elevated levels of p53 mutants, similar to those that occur in non-transformed but immortalised cell lines in the absence of contributory effects from activated oncogenes (Gjerset *et al.*,

1992). Lastly, the similar long-term survival rates of PNET patients in the faintly reactive group and the negative group suggests that in the negative group either abnormalities in genes other than p53 are involved in their development or that the p53 gene is affected by large deletions, splicing and nonsense mutations, or missense mutations outside of exons 5 to 8, as all these abnormalities can lead to loss of p53 function in the absence of p53 expression (Bodner et al., 1992).

This investigation has identified a group of PNET patients whose particularly poor response to treatment is associated with intense or high level of p53 overexpression. The high level of p53 accumulation is likely to be associated with mutations in the p53 gene, and to effect full transformation, the p53 mutants may cooperate with other oncogenes and/or tumour suppressor genes. Survival of PNET patients appears to be dependent on sensitivity of their tumours to radiation therapy (Ito et al., 1992). The poor response of PNET's expressing high levels of p53 suggests that they are less sensitive to radiotherapy than tumours showing low levels of p53 immunoreactivity. This runs counter to the previous proposals that p53 abnormalities may make tumour cells more responsive to DNA damaging agents (Lane, 1992; Vogelstein & Kinzler, 1992). However, more recently it has been demonstrated that wild type p53 is essential for the apoptic response of thymocytes to ionising radiation (Lowe et al., 1993; Clarke et al., 1993), and it has been proposed that abrogation of the p53 pathway may be involved in the poor response of many human tumours to treatment by radiation and chemotherapeutic drugs (Lane, 1993). It remains to be established whether such p53 mediated responses are relevant to PNET's. We are currently engaged in DNA sequencing studies to further elucidate the relationship between the intensity of p53 overexpression, p53 gene mutation, and survival in PNET patients.

The support of the North of England Children's Cancer Research Fund is gratefully acknowledged. The survival curves were generated and analysed by Log-Rank statistics using a programme developed by J. Smith and M. Cole from the Department of Child Health, the University of Newcastle upon Tyne. Thanks are due to Dr M. Nurbhai from the Department of Neuropathology, Middlesbrough, for providing samples from 8 PNET patients, to Mr W. McMeekin and his team from the Department of Neuropathology, Newcastle General Hospital, for cutting the sections, and to Mrs L. More from the North of England Children's and Young People's Malignant Disease Registry for supplying the information about the clinical outcome for patients.

### References

BARBARESCHI, M., IUZZOLINO, P., PENNELLA, A., ALLEGRANZA, A., ARRIGONI, G., DALLA PALMA, P. & DOGLIONI, C. (1992). p53 protein expression in central nervous system neoplasms. *J. Clin. Pathol.*, **45**, 583-586.

BARNES, D.M., HANBY, A.M., GILLETT, C.E., MOHAMMED, S., HODGSON, S., BOBROW, L.G., LEIGH, I.M., PURKIS, T., MACGEOCH, C., SPURR, N.K., BÁRTEK, J., VOJTĚŠEK, B., PICKSLEY, S.M. & LANE, D.P. (1992). Abnormal expression of wild type p53 protein in normal cells of a cancer family patient. *Lancet*, **340**, 259-263.

BIGNER, S.H., FRIEDMAN, H.S., VOGELSTEIN, B., OAKES, W.J. & BIGNER, D.D. (1990). Amplification of the c-myc gene in human medulloblastoma cell lines and xenographs. *Cancer Res.*, **50**, 2347-2350.

BODNER, S.M., MINNA, J.D., JENSEN, S.M., D'AMICO, D., CARBONE, D., MITSUDOMI, T., FEDORKO, J., BUCHHAGEN, D.L., NAU, M.M., GAZDAR, A.F. & LINNOILA, R.I. (1992). Expression of mutant p53 proteins in lung cancer correlates with the class of p53 gene mutation. *Oncogene*, **7**, 743-749.

CAPUTY, A.J., MCCULLOUGH, D.C., MANZ, H.J., PATTERSON, K. & HAMMOCK, M.K. (1987). A review of the factors influencing the prognosis of medulloblastoma. *J. Neurosurg.*, **66**, 80-87.

CLARKE, A.R., PURDIE, C.A., HARRISON, D.J., MORRIS, R.G., BIRD, C.C., HOOPER, M.L. & WYLLIE, A.H. (1993). Thymocyte apoptosis induced by p53-dependent and independent pathways. *Nature*, **362**, 849-852.

COX, D.R. (1972). Regression models and lifetables. *J. R. Stat. Soc.*, **34**, 187-220.

DE FROMENTEL, C.C. & SOUSSI, T. (1992). TP53 tumour suppressor gene: a model for investigating human mutagenesis. *Genes, Chromosomes & Cancer*, **4**, 1-15.

FINLEY, C.A., HINDS, P.W., TAN, T.H., ELIYAHU, D., OREN, M. & LEVINE, A.J. (1988). Activating mutations for transformation by p53 produce a gene product that forms an hsc 70-p53 complex with an altered half-life. *Mol. Cell. Biol.*, **8**, 531-539.

GILBERTSON, R.J., JAROS, E., PERRY, R.H. & PEARSON, A.D.J. (1992). Prognostic factors in medulloblastoma. *Lancet*, **340**, 480.

GJERSET, R.A., ARYA, J., VOLKMAN, S. & HAAS, M. (1992). Association of induction of a fully tumorigenic phenotype in murine radiation-induced T-lymphoma cells with loss of differentiation antigens, gain of CD44, and alterations in p53 protein levels. *Mol. Carcinog.*, **5**, 190-198.

GRIFFIN, C.A., HAWKINS, A.L., PACKER, R.J., RORKE, L.B. & EMANUEL, B.S. (1988). Chromosome abnormalities in paediatric tumours. *Cancer Res.*, **48**, 175-180.

HINDS, P.W., FINLAY, C.A., QUARTIN, R.S., BAKER, S.J., FEARON, E.R., VOGELSTEIN, B. & LEVINE, A.J. (1990). Mutant p53 DNA clones from human colon carcinomas cooperate with *ras* in transforming primary rat cells: A comparison of the 'hot spot' mutant phenotypes. *Cell Growth & Different.*, **1**, 571-580.

IGGO, R., GATTER, K., BÁRTEK, J., LANE, D. & HARRIS, A.L. (1990). Increased expression of mutant forms of p53 oncogene in primary lung cancer. *Lancet*, **335**, 675-679.

ITO, S., HOSHINO, T., PRADOS, M.D. & EDWARDS, M.S.B. (1992). Cell kinetics of medulloblastomas. *Cancer*, **70**, 671-678.

JAMES, C.D., HE, J., CARLBOM, E., MIKKELSEN, T., RIDDERHEIM, P.A., CAVENEE, W.K. & COLLINS, V.P. (1990). Loss of genetic information in central nervous system tumors common to children and young adults. *Genes Chromosomes & Cancer*, **2**, 94-102.

JAROS, E., PERRY, R.H., ADAM, L., KELLY, P.J., CRAWFORD, P.J., KALBAG, R.M., MENDELOW, A.D., SENGUPTA, R.P. & PEARSON, A.D.J. (1992). Prognostic implications of p53 protein, epidermal growth factor receptor, and Ki-67 labelling in brain tumours. *Br. J. Cancer*, **66**, 373-385.

KASTAN, M.B., ONYEKWERE, O., SIDRANSKY, D., VOGELSTEIN, B. & CRAIG, R.W. (1991). Participation of p53 protein in the cellular response to DNA damage. *Cancer Res.*, **51**, 6304-6311.

LANE, D.P. (1992). p53, guardian of the genome. *Nature*, **358**, 15-16.

LANE, D.P. (1993). A death in the life of p53. *Nature*, **362**, 786-787.

LODA, M., GIANGASPERO, F., BADIALI, M., CAPODIECI, P. & PESSION, A. (1992). p53 gene expression in medulloblastoma by quantitative polymerase chain reaction. *Diagn. Mol. Pathol.*, **1**, 36-44.

LOWE, S.W., SCHMITT, E.M., SMITH, S.W., OSBORNE, B.A. & JACKS, T. (1993). p53 is required for radiation-induced apoptosis in mouse thymocytes. *Nature*, **362**, 847-849.

MACGREGOR, D.N. & ZIFF, E.B. (1990). Elevated c-myc expression in childhood medulloblastomas. *Pediatr. Res.*, **28**, 63-68.

PETO, R., PIKE, M.C., ARMITAGE, P., BRESLOW, N.E., COX, D.R., HOWARD, S.V., MANTEL, N., MCPHERSON, K., PETO, J. & SMITH, P.G. (1977). Design and analysis of randomized clinical trials requiring prolonged observation of each patient. II. Analysis and examples. *Br. J. Cancer*, **35**, 1-39.

RAFFEL, C., GILLES, F.E. & WEINBERG, K.I. (1990). Reduction to homozygosity and gene amplification in central nervous system primitive neuroectodermal tumours of childhood. *Cancer Res.*, **50**, 587-591.

REICH, N.C. & LEVINE, A.J. (1984). Growth regulation of a cellular tumour antigen, p53, in nontransformed cells. *Nature*, **308**, 199-201.

REICH, N.C., OREN, M. & LEVINE, A.J. (1983). Two distinct mechanisms regulate the levels of a cellular tumour antigen, p53. *Mol. Cell. Biol.*, **3**, 2143-2150.

REIHSAUS, E., KOHLER, M., KREISS, S., OREN, M. & MONTENARCH, M. (1990). Regulation of the level of the oncoprotein p53 in non-transformed and transformed cells. *Oncogene*, **5**, 137-145.

RONEN, D., ROTTER, V. & REISMAN, D. (1991). Expression from the murine p53 promoter is mediated by factor binding to a downstream helix-loop-helix recognition motif. *Proc. Natl Acad. Sci. USA*, **88**, 4128-4132.

RORKE, L.B., GILES, F.H., DAVIS, R.L. & BECKER, L.E. (1985). Revision of the World Health Organization classification of brain tumours for childhood brain tumours. *Cancer*, **56**, 1869-1886.

RUSSELL, D.S. & RUBINSTEIN, L.J. (1989). *Pathology of Tumours of the Nervous System*. 5th ed. Edward Arnold. A division of Hodder and Stoughton: London, Melbourne, Ackland.

SIEGEL, S. & CASTELLAN, N.J. Jr. (1988). *Nonparametric Statistics for Behavioural Sciences*. 2nd ed. McGraw-Hill Book Co: New York, London.

SOLOMON, E. & BARKER, D.F. (1989). Report of the committee on the genetic constitution of chromosome 17. *Cytogenet. Cell Genet.*, **51,** 319–337.

TAIT, D.M., THORNTON-JONES, H., BLOOM, H.J., LEMERLE, J. & MORRIS-JONES, P. (1990). Adjuvant chemotherapy for medulloblastoma: the first multi-centre control trial of the International Society of Paediatric Oncology (SIOP I). *Eur. J. Cancer*, **26,** 464–469.

THOMAS, G.A. & RAFFEL, C. (1991). Loss of heterozygosity on 6q, 16q, and 17p in human central nervous system primitive neuroectodermal tumors. *Cancer Res.*, **51,** 639–643.

VOGELSTEIN, B. & KINZLER, K.W. (1992). p53 function and dysfunction. *Cell*, **70,** 523–526.

VOJTĚŠEK, B., BÁRTEK, J., MIDGLEY, C.A. & LANE, D.P. (1992). An immunochemical analysis of human p53: new monoclonal antibodies and epitope mapping using recombinant p53. *J. Immunol. Methods*, **151,** 237–244.